# Memantine, Simvastatin, and Epicatechin Inhibit 7-Ketocholesterol-induced Apoptosis in Retinal Pigment Epithelial Cells But Not Neurosensory Retinal Cells In Vitro

**DOI:** 10.18502/jovr.v15i4.7781

**Published:** 2020-10-25

**Authors:** Aneesh Neekhra, Julia Tran, Parsa R. Esfahani, Kevin Schneider, Khoa Pham, Ashish Sharma, Marilyn Chwa, Saurabh Luthra, Ana L. Gramajo, Saffar Mansoor, Baruch D. Kuppermann, M. Cristina Kenney

**Affiliations:** ^1^Gavin Herbert Eye Institute, University of California, Irvine, California; ^2^Department of Pathology and Laboratory Medicine, University of California Irvine, Irvine, CA, USA

**Keywords:** Epicatechin, 7-Ketocholesterol, Memantine

## Abstract

**Purpose:**

7-ketocholesterol (7kCh), a natural byproduct of oxidation in lipoprotein deposits is implicated in the pathogenesis of diabetic retinopathy and age-related macular degeneration (AMD). This study was performed to investigate whether several clinical drugs can inhibit 7kCh-induced caspase activation and mitigate its apoptotic effects on retinal cells in vitro.

**Methods:**

Two populations of retinal cells, human retinal pigment epithelial cells (ARPE-19) and rat neuroretinal cells (R28) were exposed to 7kCh in the presence of the following inhibitors: Z-VAD-FMK (pan-caspase inhibitor), simvastatin, memantine, epicatechin, and Z-IETD-FMK (caspase-8 inhibitor) or Z-ATAD-FMK (caspase-12 inhibitor). Caspase-3/7, -8, and -12 activity levels were measured by fluorochrome caspase assays to quantify cell death. IncuCyte live-cell microscopic images were obtained to quantify cell counts.

**Results:**

Exposure to 7kCh for 24 hours significantly increased caspase activities for both ARPE-19 and R28 cells (*P* < 0.05). In ARPE cells, pretreatment with various drugs had significantly lower caspase-3/7, -8, and -12 activities, reported in % change in mean signal intensity (msi): Z-VAD-FMK (48% decrease, *P* < 0.01), memantine (decreased 47.8% at 1 µM, *P* = 0.0039 and 81.9% at 1 mM, *P* < 0.001), simvastatin (decreased 85.3% at 0.01 µM, *P* < 0.001 and 84.8% at 0.05 µM, *P* < 0.001) or epicatechin (83.6% decrease, *P* < 0.05), Z-IETD-FMK (68.1% decrease, *P* < 0.01), and Z-ATAD-FMK (47.7% decrease, *P* = 0.0017). In contrast, R28 cells exposed to 7kCh continued to have elevated caspase-3/7, -8, and -12 activities (between 25.7% decrease and 17.5% increase in msi, *P* > 0.05) regardless of the pretreatment.

**Conclusion:**

Several current drugs protect ARPE-19 cells but not R28 cells from 7kCh-induced apoptosis, suggesting that a multiple-drug approach is needed to protect both cells types in various retinal diseases.

##  INTRODUCTION

Apoptosis is a highly regulated process of programmed cell death critical in various disease states and degenerations. These pathways are implicated in various neurodegenerative diseases (e.g., Alzheimer's disease, Parkinson's disease, amyotrophic lateral sclerosis), ocular diseases (e.g., glaucoma, diabetic retinopathy and age-related macular degeneration [AMD]), and immunologic disorders.^[1,2,3,4,5,6]^ While treatments for the wet form of AMD rely on inhibiting choroidal neovascularization via anti-vascular endothelial growth factor (anti-VEGF) injections,^[7]^ there is currently no cure for the dry form of AMD.

A pro-apoptotic oxysterol implicated in AMD is 7-ketocholesterol (7kCh), a toxic metabolite generated from the oxidation of cholesterol-esters in low density lipoprotein (LDL), atheromatous plaques, and drusen.^[6,8,9,10]^ 7kCh activates three distinct kinase signaling pathways via NFkB, MAPK, and ERK to upregulate pro-inflammatory cytokines: IL-6, IL-8, and vascular endothelial growth factor (VEGF) which induce neovascularization in the choroid.^[7,8,11,12,13]^ In previous studies, retinal pigment epithelial (RPE) cells and vascular endothelial cells had an 8- to 10-fold increase in VEGF levels,^[14]^ and a 2-fold increase in endothelial smooth muscle cells after exposure to 7kCh.^[15]^ Our previous studies showed that in both ARPE-19 and rat neuroretinal R28 cell cultures, 7kCh significantly increased the levels of pro-apoptotic caspase-3, -8, and -12 activities.^[16,17,18,19]^ 7kCh is also a chemoattractant that directs microglia to the outer retina to produce metalloproteinases that cause breaks in Bruch's membrane and produce pro-angiogenic substances.^[11,14,20]^ A 2019 study showed that 7kCh intravitreally injected into rat retinas induced apoptosis in photoreceptors and RPE cells and caused microvilli detachment in the outer segment.^[10]^ Thus, the toxic accumulation of 7kCh over decades may activate cellular processes that predispose patients to age-related ocular pathologies such as AMD.^[8]^

Despite the strong evidence of 7kCh's role in age-related diseases, little is known about its detoxifying mechanisms. A 2019 study showed that compounds such as vitamin E, oleic acid, terpenoids, and polyphenols inhibit 7kCh-induced apoptosis,^[21]^ while resveratrol, an antioxidant and anti-inflammatory stilbenoid found in the skin of grapes and red wine inhibits 7kCh-induced VEGF expression.^[22]^ Successful inhibition by these natural compounds advances our knowledge related to functional food therapies.^[21]^

Thus, it is necessary to explore the potential drugs or inhibitors given the limited body of knowledge on 7kCh detoxification. The present study investigates six different inhibitors (simvastatin [lipid lowering medication], memantine [used to treat Alzheimer's disease], epicatechin [flavonoid present in green tea], Z-VAD-FMK [pan-caspase inhibitor], Z-IETD-FMK [caspase-8 inhibitor], and Z-ATAD-FMK [caspase-12 inhibitor]) to identify drugs/agents that can block the 7kCh-induced caspase activation. Our results show that these drugs are effective in ARPE-19 cells but not in R28 cells, implicating the need for a multidrug approach or novel therapies to inhibit 7kCh in various cell types.

##  METHODS

### Cell Culture

R28 rat neurosensory cells have properties that resemble those of various human neurosensory cells. The R28 cells derived from retina of post-natal day 6 rats were cultured in Dulbecco's modified Eagle's media, 10µg/mL gentamicin, 10mM non-essential amino acids, and high glucose (DMEM high glucose; Invitrogen-Gibco, Carlsbad, CA) with 10% fetal bovine serum. Despite their clonal origin, R28 cells are characteristically heterogeneous, display both glial and neuronal cell markers, and have functional neuronal properties.^[23]^ R28's diversity of cell types, ability to respond to a variety of stimuli, and differentiation potential makes it an appropriate model for neuroretinal tissue.^[24]^

A 1:1 mixture (vol/vol) of Dulbecco's modified Eagle's and Ham's nutrient mixture F-12 was used to grow ARPE-19 cells (ATCC, Manassas, VA); (Invitrogen-Gibco, Carlsbad, CA), 0.37% sodium bicarbonate, 0.058% L-glutamine, 10% fetal bovine serum, antibiotics (100U/ml penicillin G, 0.1mg/ml streptomycin sulfate, 10µg/mL gentamicin), 10mM non-essential amino acids, and an anti-fungal agent (amphotericin-B 2.5µg/mL). ARPE-19 cells are a homogeneous, retinal-derived cell line with functional and structural properties similar to human RPE cells.^[25]^ All cells within the ARPE-19 culture express RPE-specific markers such as CRALBP, BEST1, and RPE-65.^[26]^

### Cell Plating

Cells were plated on 24 well plates (Becton Dickinson Labware, Franklin Lakes, NJ) at 150,000 cells per well and incubated at 37°C in 5% CO${}_{2}$ until confluent. Cell incubation was performed in a serum-free media for 24 hours to prevent excessive proliferation. Cells in the experimental group were then pretreated with various inhibitors while the cells in the control group were only exposed to DMSO (the vehicle for 7kCh). All experimental cells besides the control cells were then exposed to 30 µg/ml 7kCh for 24 hours before caspase activity was assessed.

### Caspase-3/7, -8, and -12 Detection 

Carboxyfluorescein FLICA Caspase Apoptosis Detection kits (Immunochemistry Technologies LLC, Bloomington, MN) were used to quantify cells undergoing caspase-mediated apoptosis. The FLICA Detection Reagent contains a caspase inhibitor sequence linked to a fluorescent carboxyfluorescein probe with an optimal excitation range of 488–492 nm and an emission range of 515–535 nm. The probe only fluoresces when the FLICA reagent covalently binds to activated caspase-3/7 and -8 *in vitro*. The fluorescence intensity quantifies the number of whole, living cells undergoing caspase-mediated apoptosis since cells with premature, inactivated, or no caspase activity will not fluoresce. This was measured using the Fluorescence Image Scanning Unit (FMBIO III) instrument (Hitachi, Yokohama, Japan).

Caspase-12 activity was detected using CaspGLOW Fluorescein Caspase-12 staining kit (BioVision). The assay utilizes the caspase-12 inhibitor, Z-ATAD-FMK (Ala-Thr-Ala-Asp-fluoromethylketone), conjugated to FITC (FITC-ATAD-FMK) as a marker. FITC-ATAD-FMK irreversibly binds to activated caspase-12 in cells undergoing apoptosis and is cell permeable and nontoxic.

Fresh culture media were used to rinse the wells at 24 hours. Then, 300 µl/well of FLICA solution in culture media were placed in the wells for 1 hour. Phosphate buffered saline (PBS) was used to wash the wells three times. The experimental groups were (1) R28 and ARPE-19 cells with 30 µg/ml 7kCh alone; (2) R28 and ARPE-19 cells with inhibitor; and (3) R28 and ARPE-19 cells with DMSO. The different inhibitors used were 1 µM and 1 mM memantine (Alexis Biochemical, San Diego), 0.01 µM and 0.05 µM simvastatin (Merck and Co., Whitehouse Station, NJ), 5 µM epicatechin (Sigma Aldrich Inc, St Louis, MO), the pan-caspase inhibitor Z-VAD-FMK (Val-Ala-Asp-fluoromethylketone, Immunochemistry Technologies LLC, Bloomington, MN), the caspase-8 inhibitor (Z-IETD-FMK, Iso-Glu-Thr-Asp-fluoromethylketone, Calbiochem, La Jolla, CA), and caspase-12 inhibitor (Z-ATAD-FMK, Ala-Thr-Ala-Asp-fluoromethylketone, Calbiochem, La Jolla, CA). The inhibitors were added to the culture medium 1 hour before exposing cells to 7kCh.

In addition to the experimental groups, we also analyzed the following control groups: (1) untreated R28 cells and ARPE-19 cells without FLICA or CaspGLOW to exclude autofluorescence from cells; (2) untreated R28 and ARPE-19 cells with FLICA or CaspGLOW for comparison of caspase activity of treated cells; (3) tissue culture plate wells (without cells) with buffer alone to represent the background levels; (4) tissue culture plate wells without cells with culture media and DMSO in order to exclude cross-reaction of culture media and/or DMSO with the plastic material of the tissue culture plate or cross-reaction of FLICA or CaspGLOW; (5) R28 cells and ARPE-19 cells with DMSO and FLICA to account for any cross-fluorescence due to the cross-reaction of untreated cells with DMSO; and (6) untreated R28 and ARPE-19 cells with negative control Z-FA-FMK and FLICA as a control for the pan caspase inhibitor.

IncuCyteⓇ Live-Cell Imaging Analysis was used to capture fluorescent images in real time. NucLight Rapid Red and Caspase-3/7 Green reagents were used to stain cells in 96-well plates at 5,000–10,000 cells/well. The NucLight Rapid Red reagent quantifies the amount of cell proliferation in IncuCyte live-cell images by staining the nuclei of living cells. The Caspase-3/7 Green reagent contains an oligopeptide cleavage sequence (DEVD) conjugated to a DNA-binding dye. The green reagent labels apoptotic cells at an emission maximum of 533 nm once the sequence is cleaved by caspase-3/7. Images were captured per well every 3 hours for five days with Brightfield, Red, and Green channels. Apoptosis due to caspase-3/7 activity is discriminated from live and necrotic cells by overlapping the signal counts from NucLight Red with the Caspase-3/7 green.

### Statistical Analysis

ANOVA using GraphPad Prism 3.0 version statistics program (GraphPad Software Inc., San Diego, CA) was used for statistical analysis of the data. The data within each experiment were compared using the Newman–Keuls multiple comparison test. Mean ± standard error of mean (SEM) was used to present the data. Experiments were performed in triplicate. *P-*values < 0.05 were considered statistically significant.

##  RESULTS

### Pan-caspase Inhibitor Z-VAD-FMK Reduces Caspase-3/7 Activity in Only ARPE-19 Cells 

**Figure 1:**
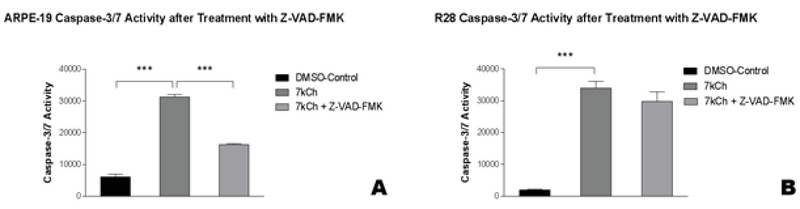
Caspase-3/7 activity in 7kCh-treated ARPE-19 and R28 cells in response to 7kCh alone or pretreatment with Z-VAD-FMK. Cells exposed to 7kCh alone increased caspase-3/7 activity*** (A–B). ARPE-19 cells pretreated with Z-VAD-FMK decreased caspase-3/7 activity*** (A). In contrast, R28 cells pretreated with Z-VAD-FMK did not have decreased caspase-3/7 activity (B). *P < 0.05, **P < 0.01, ***P < 0.001. The error bars represent the standard error of the mean (SEM) of measurements for the three conditions in three separate runs (n = 9, *A* *–* *B* *).*

ARPE-19 cells exposed to 7kCh had increased caspase-3/7 activity (31,311 ± 766.7 msi, *P* < 0.0001) compared to the ARPE-19 cells exposed only to DMSO (6,086 ± 910 msi, Figure 1A). Caspase-3/7 activity in ARPE-19 cells decreased significantly at 48.0% following pretreatment with Z-VAD-FMK (16,278 ± 323.1 msi, *P* < 0.0001, Figure 1A). R28 cells exposed to 7kCh had increased caspase-3/7 activity (34,014 ± 2,126 msi, *P* = 0.0001) compared to the R28 cells exposed only to DMSO (1,975 ± 234.5 msi, Figure 1B). Caspase-3/7 activity in R28 cells did not decrease significantly at 12.3% following pretreatment with Z-VAD-FMK (29,805 ± 3,007 msi, *P* > 0.05, Figure 1B). The pan-caspase inhibitor Z-VAD-FMK was not able to protect the R28 cells.

**Figure 2:**
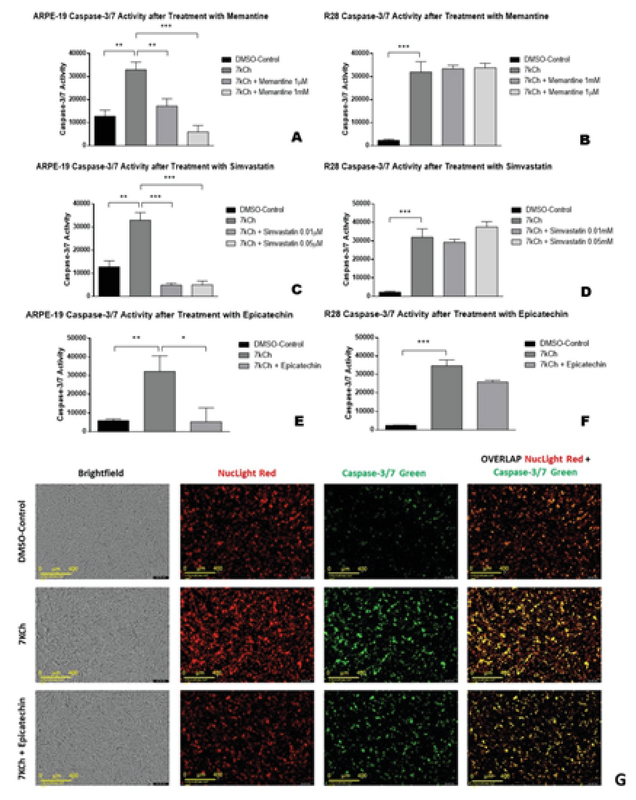
Caspase-3/7 activity in 7kCh-treated ARPE-19 and R28 cells after pretreatment with memantine, simvastatin, or epicatechin. All cells exposed to 7kCh alone increased caspase-3/7 activity (A–F). Pretreatment of ARPE-19 cells with 1 µM** and 1 mM*** memantine (A) 0.01 µM*** and 0.05 µM*** simvastatin (C), or 5 µM epicatechin* (E) showed decreased caspase-3/7 activity. In contrast, R28 cells pretreated with 1 µM and 1 mM memantine (B) 0.01 µM and 0.05 µM simvastatin (D), or 5 µM epicatechin (F) did not have significantly decreased caspase-3/7 activity. Representative IncuCyte live-cell images of DMSO-Control, 7kCh, and 7kCh with epicatechin-treated ARPE-19 cells at 24 hours (G). There are no major differences among treatments detected with brightfield microscopy (G, first column). ARPE-19 cells stressed with 7kCh demonstrate increased nuclear staining number, increased caspase-3/7 signal number, and an increased overlap signal number (G, second row). These counts decrease when ARPE-19 cells are pretreated with epicatechin before 7kCh (G, third row). *P < 0.05, **P < 0.01, ***P < 0.001. The error bars represent the standard error of the mean (SEM) of measurements for the four conditions in three separate runs (n = 12, *A* *–* *D* *) and three conditions in three separate runs (n = 9,* *E* *–* *F* *). Scale bar = 400 μM (* *G* *). *

**Figure 3:**
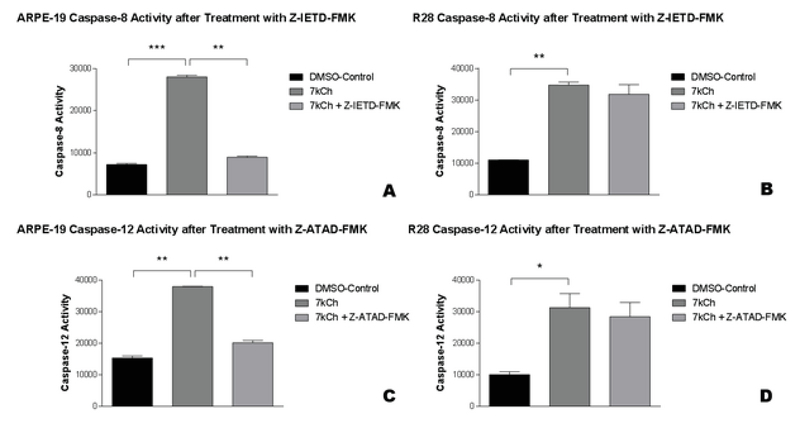
Caspase-8 and -12 activity in 7kCh-treated ARPE-19 and R28 cells in response to 7kCh alone or pretreatment with Z-IETD-FMK or Z-ATAD-FMK. All cells exposed to 7kCh alone showed increased caspase-3/7 activity (A–F). ARPE-19 cells pretreated with Z-IETD-FMK** (A) or Z-ATAD-FMK** (C) showed decreased caspase-8 and -12 activity, respectively. In contrast, R28 cells pretreated with Z-IETD-FMK (B) or Z-ATAD-FMK (D) did not show decreased caspase-8 nor -12 activities, respectively. *p < 0.05, **p < 0.01, ***p < 0.001. The error bars represent the standard error of the mean (SEM) of measurements for the three conditions in three separate runs (n = 9, *A* *–* *D* *).*

### 1 µM and 1 mM Memantine Reduce Caspase-3/7 Activity in Only ARPE-19 Cells

ARPE-19 cells exposed to 7kCh had increased caspase-3/7 activity (32,972 ± 1,891 msi, *P* = 0.001) compared to ARPE-19 cells exposed only to DMSO (12,791 ± 1,501 msi, Figure 2A). Caspase-3/7 activity in ARPE-19 cells decreased significantly at 47.8% following pretreatment with 1 µM memantine (17,218 ± 1,835 msi, *P* = 0.003, Figure 2A) and even more so at 81.9% with 1 mM memantine (5,969 ± 1,596 msi, *P* < 0.001, Figure 2A). R28 cells exposed to 7kCh had increased caspase-3/7 activity (31,974 ± 2,599 msi, *P* < 0.001) compared to R28 cells exposed only to DMSO (2,355 ± 254.4 msi, Figure 2B). Caspase-3/7 activity in R28 cells did not decrease significantly following pretreatment with 1 µM memantine (33,850 ± 1,894 msi, *P* > 0.05, Figure 2B) nor with 1 mM memantine (33,422 ± 863.7 msi, *P* > 0.05, Figure 2B). Neither concentration of the NMDA inhibitor memantine protected the R28 cells from apoptosis.

### 0.01 µM and 0.05 µM Simvastatin Reduce Caspase-3/7 Activity in Only ARPE-19 Cells

ARPE-19 cells exposed to 7kCh had increased caspase-3/7 activity (32,972 ± 1,891 msi, *P* = 0.001) compared to ARPE-19 cells exposed only to DMSO (12,791 ± 1,501 msi, Figure 2C). Caspase-3/7 activity in ARPE-19 cells decreased significantly at 85.3% following pretreatment with 0.01 µM simvastatin (4,849 ± 500.2 msi, *P* < 0.001, Figure 2C) and at 84.8% with 0.05 µM simvastatin (5,016 ± 968.5 msi, *P* < 0.001, Figure 2C). R28 cells exposed to 7kCh had increased caspase-3/7 activity (31,974 ± 2,599 msi, *P* < 0.001) compared to R28 cells exposed only to DMSO (2,355 ± 254.4 msi, Figure 2D). Caspase-3/7 activity in R28 cells did not decrease significantly at 8.3% following pretreatment with 0.01 µM simvastatin (29,316 ± 897.9 msi, *P* > 0.05, Figure 2D) nor with 0.05 µM simvastatin, which had a slight increase in activity at 17.5% (37,575 ± 1,629 msi, *P* > 0.05, Figure 2D). Neither concentration of simvastatin protected the R28 cells from apoptosis.

### 5 µM Epicatechin Reduces Caspase-3/7 Activity in Only ARPE-19 Cells

ARPE-19 cells exposed to 7kCh had increased caspase-3/7 activity (32,181 ± 4,839 msi, *P* = 0.005, Figure 2E) compared to ARPE-19 cells exposed only to DMSO (5,936 ± 491.4 msi, Figure 2E). Caspase-3/7 activity in ARPE-19 cells decreased significantly at 83.6% following pretreatment with 5 µM epicatechin (5,263 ± 4,344 msi, *P* < 0.05, Figure 2E). R28 cells exposed to 7kCh had increased caspase-3/7 activity (34,720 ± 3,197 msi, *P* < 0.001, Figure 2F) compared to R28 cells exposed only to DMSO (2,488 ± 116.3 msi, Figure 2F). Caspase-3/7 activity in R28 cells decreased at 25.7% following pretreatment with 5 µM epicatechin (25,806 ± 977.5 msi, *P* = 0.05, Figure 2F), but the *p*-value was not significant.

Representative IncuCyte live-cell images of ARPE-19 cells treated with DMSO-Control, 7kCh, and 7kCh with epicatechin, respectively, are shown in Figure 2G. 7kCh increased NucLight Red signal, Caspase-3/7 green signal, and overlap signal counts at 24 hours (Figure 2G, second row) compared with DMSO-Control (Figure 2G, first row) in ARPE-19 cells. These counts are reduced when cultures are pretreated with inhibitors such as epicatechin before exposure to 7kCh (Figure 2G, third row).

### Caspase-8 Inhibitor Z-IETD-FMK Reduces Caspase-8 Activity in Only ARPE-19 Cells

ARPE-19 cells exposed to 7kCh had increased caspase-8 activity (28,037 ± 398 msi, *P* < 0.001, Figure 3A) compared to ARPE-19 cells exposed only to DMSO (7,210 ± 478.8 msi, Figure 3A). Caspase-8 activity in 7kCh exposed ARPE-19 cells was significantly reduced at 68.1% following pretreatment with Z-IETD-FMK (8,952 ± 283 msi, *P* < 0.01, Figure 3A). R28 cells exposed to 7kCh had increased caspase-8 activity (34,734 ± 944.5 msi, *P* = 0.001, Figure 3B) compared to R28 cells exposed only to DMSO (11,043 ± 55.5 msi, Figure 3B). Caspase-8 activity in 7kCh-exposed R28 cells was not significantly reduced at 8.4% by pretreatment with Z-IETD-FMK (31,825 ± 3,073, *P* > 0.05, Figure 3B).

### Caspase-12 Inhibitor Z-ATAD-FMK Reduces Caspase-12 Activity in Only ARPE-19 Cells

ARPE-19 cells exposed to 7kCh had increased caspase-12 activity (38,585 ± 1,804 msi, *P* < 0.001, Figure 3C) compared to ARPE-19 cells exposed only to DMSO (15,351 ± 636.5 msi, Figure 3C). Caspase-12 activity in 7kCh-exposed ARPE-19 cells was significantly reduced at 47.7% by pretreatment with Z-ATAD-FMK (20,175 ± 719 msi, *P* = 0.001, Figure 3C). R28 cells exposed to 7kCh had an increased caspase-12 activity (31,234 ± 4,445 msi, *P* < 0.05, Figure 3D) compared to R28 cells exposed only to DMSO (10,043 ± 944.5 msi, Figure 3D). Caspase-12 activity in 7kCh-exposed R28 cells was not significantly reduced at 9.1% by pretreatment with Z-ATAD-FMK (28,379 ± 4518 msi, *P* > 0.05, Figure 3D).

##  DISCUSSION

This study emphasized 7kCh's role in caspase activation and highlighted several clinical drugs that protected RPE cells but not R28 cells from 7kCh-induced apoptosis. Research in the past two decades has strongly supported 7kCh's role in AMD pathogenesis. 7kCh from LDL is esterified via two enzymes, cPLA2α and SOAT1, to 7kCh-fatty acid esters (7KFAEs), and effluxed by HDL to the liver for bile acid production.^[27]^ Cholesterol levels may therefore contribute to ocular disease as high serum levels of LDL or 7KFAEs (which revert to 7kCh), or low levels of HDL can result in increased 7kCh levels.^[27]^ 7kCh may also accumulate over time from natural mechanisms such as the rhodopsin cycle and other photo-oxidative processes.^[28]^ Compared to controls, 7kCh was found at 6- to 50-fold higher levels in RPE and photoreceptor inner segments of photodamaged rat retinas,^[9]^ and at 4-fold levels in Bruch's membrane and neuroretinal cells in photodamaged monkey retinas.^[14,28]^ Excess 7kCh can trigger caspase pathways, excess reactive oxygen species (ROS) production, endothelial dysfunction, breaks in Bruch's membrane, induction of cytokines IL-6 and IL-8, and neovascularization in the choroid ^[16,17,18,19][20][29][30][31]^, which are all consistent with AMD. Additional damage includes increased levels of DNA fragmentation and increased chromatin condensation in both ARPE-19 and R28 cells.^[32]^ 7kCh also damages mitochondria by inducing reactive oxygen/nitrogen species, reducing the mitochondrial membrane potential by 2.2-fold (*P* < 0.001), and decreasing levels of intact 16.2-kb mtDNA.^[16]^

The clinically available inhibitors used in this study all prevented 7kCh induced caspase-3/7 activation in ARPE-19 cells but not R28 cells:

(1) Memantine is a moderate-affinity, noncompetitive antagonist of the glutamatergic N-methyl-D-aspartate (NMDA) receptors. It suppresses glutamate-excitotoxicity in animal models associated with neurological diseases such as Alzheimer's, vascular dementia, and retinal ganglion cell death after ischemic strokes.^[33,34,35,36,37]^ By inhibiting downstream p53 and calpain-caspase-3, it reduces Ca${}^{2+}$-dependent production of NO and O2(–) and halts neuronal apoptosis in rats with middle cerebral artery occlusion.^[36,38,39]^ A previous study showed that memantine reduces caspase-3/7 activity in staurosporine-treated neuronal cells.^[40]^ The present study showed 1 μM and 1 mM of memantine reduced caspase-3/7 activity by 47.8% and 81.9% in ARPE-19 cells, respectively, but only 5.9% and 4.5% in R28 cells (Figures 2A–2B) despite evidence of immunoreactivity to NMDA and GABAa receptors.^[41]^ This may be because memantine can upregulate brain-derived neurotrophic factor through the PI3-K/Akt pathway independent of NMDA receptors.^[42]^ Furthermore, R28 cells derived from six-day old rat retina are not fully differentiated and may not have well-developed NMDA receptors. R28 cultures are also heterogeneous and memantine may preferentially affect neural cells with NMDA receptors over non-neural glial cells.^[23]^

(2) Simvastatin is a hydrophobic drug that inhibits 3-Hydroxy-3-methyl-glytaryl coenzyme A (HMG-CoA) reductase and is frequently utilized to reduce serum LDL in hyperlipidemic patients.^[43]^ Besides its lipid-lowering effects, it is also neuroprotective and upregulates Bcl-2, a major cell survival protein,^[44,45]^ and induces Hsp27, a heat shock protein that promotes retinal ganglion cell survival.^[46]^ In rats with streptozotocin-induced diabetes, therapeutic levels of simvastatin slowed progression of diabetic retinopathy by reducing VEGF activation, vascular permeability, and endothelial-leukocyte adherence.^[47]^ The effects of statins are notably biphasic: low concentrations of statins are pro-angiogenic, while high concentrations are angiostatic.^[48,49,50]^ Higher concentrations can also induce caspase-3/7 apoptosis in human monocytes, pericytes, and tumor cells.^[50,51,52]^ Our results show that 0.01 μM and 0.05 μM of simvastatin reduced caspase-3/7 activity by 85.3% and 84.8%, respectively, in ARPE-19 cells (Figure 2C), while 0.05 μM simvastatin increased caspase activity by 17.5% in R28 cells (Figure 2D). The therapeutic dose in ARPE-19 cells was toxic in R28 cells, suggesting underdeveloped drug import/export systems or excess HMG-CoA reductase concentrations in R28 cells, but further studies are needed.

(3) Epicatechin is a powerful antioxidant flavanol found in many plant-based foods such as grapes, dark chocolate, and green tea. It is commonly used to reduce oxidative damage by inhibiting nicotinamide adenine dinucleotide phosphate (NADPH) oxidase and maintaining nitric oxide (NO) synthase, which prevents oxidized LDL endothelial dysfunction implicated in dementia.^[53,54,55]^ It can also decrease levels ROS in the hippocampus of rat models with hypertension and Alzheimer's.^[56]^ Other studies show that epigallocatechin gallate, a derivative with phenolic hydroxyl, injected into rat retinas attenuated sodium nitroprusside-induced oxidative stress in retinal ganglion cells.^[57]^ Our results show that epicatechin significantly reduced caspase-3/7 activity by 83.6% in ARPE-19 cells, but only 25.7% in R28 cells (*P* = 0.05. Figures 2E—2F). Unlike the first two inhibitors, epicatechin's borderline *p*-value suggests it protects R28 cells by decreasing general oxidative burden. Thus, targeting upstream ROS production via NADPH oxidase may protect multiple retinal cell types more effectively than targeting specific downstream enzymes or receptors. These results are consistent with those obtained by IncuCyteⓇ Live-Cell Imaging Analysis which captures caspase-3/7-mediated apoptosis in real-time images (Figure 2G). The overlapped signal (Figure 2G, fourth column) shows increased caspase activity with 7kCh (Figure 2G, second row) that was reduced with epicatechin pretreatment (Figure 2G, third row). Figure 2G shows the representative images of ARPE-19 cells treated with DMSO-control (first row), 7kCh (second row), and pretreated with epicatechin (third row). Similar image results were obtained with ARPE-19 cells pretreated with simvastatin or memantine.

(4) Z-VAD-FMK is a direct pan-caspase inhibitor that irreversibly binds the catalytic site of various caspase proteases. It prevented cell shrinkage and DNA fragmentation due to caspase-2, -3, -6, and -8 in flounder immune cells,^[58]^ and prevented increases in p53, PARP-1, and caspase-3 levels after 35 hours of glucose deprivation in retinal ganglion cells.^[59]^ In ARPE-19 cells, the Z-VAD-FMK reduced 7-kCh-induced caspase-3/7 activity by 48.0% but did not protect R28 (12.3%).

(5) Z-IETD-FMK is a direct caspase-8 inhibitor that disrupts the extrinsic caspase pathway. It reduced caspase-8 activity by 68.1% in ARPE-19 cells but had no protective effects in neuroretinal cells (8.4%).

(6) Z-ATAD-FMK is a caspase-12 inhibitor that disrupts the endoplasmic reticulum stress-induced caspase pathway. In ARPE-19 cells, it reduced caspase-12 activity by 47.7%. In contrast, there was no significant effect on R28 cells, with only 9.1% decrease in caspase activity.

Previous studies demonstrated that a component of cigarette smoke, benzo(e)pyrene [B(e)P], also induces caspase-3/7 activity in ARPE-19 cells and was inhibited by genistein, resveratrol, and memantine but not calpain, BTIC, simvastatin, or epicatechin.^[60]^ When combined with the current study's results, memantine could reverse both B(e)P- and 7kCh-induced caspase-3/7 activities in ARPE-19 cells, but simvastatin and epicatechin only reversed activity induced by 7kCh not B(e)P. This indicates that the same cell type can have distinct pathways to activate and inhibit caspase-3/7, depending on the insult.

Likewise, the current study suggests that different cell types have distinct pathways to activate and inhibit apoptosis induced by 7kCh. None of the drugs significantly reduced caspase activity in R28 cells, which strongly implies that tissue characteristics or processes maintain apoptosis in the presence of inhibitors. As mentioned previously, R28 cells are retinal precursor cells from six-day old rats that express genes and proteins specific to their developmental age, which may not include receptors that respond to the drugs.^[41]^ Furthermore, this heterogeneous population has cell types with distinct morphologies, biochemical characteristics, and neuronal-specific cell markers. A subset of R28 cells have receptors to neurotransmitters such as dopamine, serotonin, glycine, and acetylcholine; other cells have receptors that respond to NMDA and GABA agonists; still others show immunoreactivity to GluR1, GluR2, and GluR3.^[24,41]^ In contrast, ARPE-19 cells are a homogeneous population of RPE cells that grow in a monolayer, express the same receptors and RPE-selective markers like CRALBP, and have the same cobblestone morphology.^[61]^ Thus, receptor- or enzyme-specific drugs such as the caspase inhibitors, memantine, or simvastatin will affect all ARPE-19 cells equally and the global response is additive (–47.7% to –85.3% change in caspase activity, all *p*-values < 0.05). In contrast, only a fraction of R28 cells that express the target enzyme or receptor will be affected, and the global response is represented only by the subset (+17.5% to –25.7% change in caspase activity, all *p*-values > 0.05).

In summary, the current study quantified caspase-3/7 activity as a marker of apoptosis between human ARPE-19 cells and rat R28 cells after treatment with 7kCh and various inhibitors. 7kCh significantly induced caspase-3/7 for both cultures, yet pretreatment with anti-apoptotic drugs (memantine, simvastatin, epicatechin, Z-VAD-FMK, etc.) consistently reduced caspase activities only in the ARPE-19 cells. This discrepancy in caspase deactivation between ARPE-19 and R28 cells may be related to the differences in the heterogeneity of the cell population or the age and species of the original tissue. Ultimately, the etiology of AMD is multifactorial and future treatments may utilize combination therapy to inhibit common toxins, like 7kCh and B(e)P, that affect multiple retinal cell targets.

##  Financial Support and Sponsorship

This work was supported by the Discovery Eye Foundation, Polly and Michael Smith, Edith and Roy Carver, and NEI R01 EY0127363 (MCK and in part by an Unrestricted Departmental Grant from Research to Prevent Blindness. The authors are also thankful to the Institute for Clinical and Translational Science (ICTS) at University of California Irvine.

##  Conflicts of Interest

There are no conflicts of interest.

## References

[1] Favaloro B, et al. Role of apoptosis in disease. *Aging* 2012;4:330–349. 10.18632/aging.100459 PMC3384434 22683550

[2] Hinton DR, He S, Lopez PF. Apoptosis in surgically excised choroidal neovascular membranes in age-related macular degeneration. *Arch Ophthalmol* 1998;116:203–209. 10.1001/archopht.116.2.203 9488273

[3] Obulesu M, Lakshmi MJ. Apoptosis in Alzheimer's disease: an understanding of the physiology, pathology and therapeutic avenues. *Neurochem Res* 2014;39:2301–2312. 10.1007/s11064-014-1454-4 25322820

[4] Samadi A, et al. Oxysterol species: reliable markers of oxidative stress in diabetes mellitus. *J Endocrinol Invest* 2019;42:7–17. 10.1007/s40618-018-0873-5 29564756

[5] Thomas CN, et al. Caspases in retinal ganglion cell death and axon regeneration. *Cell Death Discov* 2017;3:17032. 10.1038/cddiscovery.2017.32 PMC5903394 29675270

[6] Xu GZ, Li WW, Tso MO. Apoptosis in human retinal degenerations. *Trans Am Ophthalmol Soc* 1996;94:411–430; discussion 430–431. PMC1312106 8981707

[7] Wei Q, et al. Combination of bevacizumab and photodynamic therapy vs. bevacizumab monotherapy for the treatment of wet age-related macular degeneration: A meta-analysis of randomized controlled trials. *Exp Ther Med* 2018;16:1187–1194. 10.3892/etm.2018.6305 PMC6090202 30116368

[8] Pariente A, et al. Inflammatory and cell death mechanisms induced by 7-ketocholesterol in the retina. Implications for age-related macular degeneration. *Exp Eye Res* 2019;187:107746. 10.1016/j.exer.2019.107746 31394101

[9] Rodriguez IR, Larrayoz IM. Cholesterol oxidation in the retina: implications of 7KCh formation in chronic inflammation and age-related macular degeneration. *J Lipid Res* 2010;51:2847–2862. 10.1194/jlr.R004820 PMC2936760 20567027

[10] Yang C, et al. 7-Ketocholesterol disturbs RPE cells phagocytosis of the outer segment of photoreceptor and induces inflammation through ERK signaling pathway. *Exp Eye Res* 2019;189:107849. 10.1016/j.exer.2019.107849 31655042

[11] Indaram M, et al. 7-Ketocholesterol increases retinal microglial migration, activation, and angiogenicity: a potential pathogenic mechanism underlying age-related macular degeneration. *Sci Rep* 2015;5:9144. 10.1038/srep09144 PMC4360733 25775051

[12] Larrayoz IM, et al. 7-ketocholesterol-induced inflammation: involvement of multiple kinase signaling pathways via NFkappaB but independently of reactive oxygen species formation. *Invest Ophthalmol Vis Sci* 2010;51:4942–4955. 10.1167/iovs.09-4854 PMC3066624 20554621

[13] Wang H, et al. Thy-1 Regulates VEGF-Mediated Choroidal Endothelial Cell Activation and Migration: Implications in Neovascular Age-Related Macular Degeneration. *Invest Ophthalmol Vis Sci* 2016;57:5525–5534. 10.1167/iovs.16-19691 PMC5080948 27768790

[14] Moreira EF, et al. *7*-Ketocholesterol is present in lipid deposits in the primate retina: potential implication in the induction of VEGF and CNV formation. *Invest Ophthalmol Vis Sci* 2009;50:523–532. 10.1167/iovs.08-2373 PMC2811433 18936140

[15] Dulak J, et al. Vascular endothelial growth factor synthesis in vascular smooth muscle cells is enhanced by 7-ketocholesterol and lysophosphatidylcholine independently of their effect on nitric oxide generation. *Atherosclerosis* 2001;159:325–332. 10.1016/s0021-9150(01)00520-2 11730812

[16] Gramajo AL, et al. Mitochondrial DNA damage induced by 7-ketocholesterol in human retinal pigment epithelial cells in vitro. *Invest Ophthalmol Vis Sci* 2010;51:1164–1170. 10.1167/iovs.09-3443 19834037

[17] Luthra S, et al. 7-Ketocholesterol activates caspases-3/7, -8, and -12 in human microvascular endothelial cells in vitro. *Microvasc Res* 2008;75:343–350. 10.1016/j.mvr.2007.10.003 18068200

[18] Luthra S, et al. Activation of caspase-8 and caspase-12 pathways by 7-ketocholesterol in human retinal pigment epithelial cells. *Invest Ophthalmol Vis Sci* 2006;47:5569–5575. 10.1167/iovs.06-0333 17122150

[19] Neekhra A, et al. Caspase-8, -12, and -3 activation by 7-ketocholesterol in retinal neurosensory cells. *Invest Ophthalmol Vis Sci* 2007;48:1362–1367. 10.1167/iovs.06-0900 17325185

[20] Javitt NB, Javitt JC. The retinal oxysterol pathway: a unifying hypothesis for the cause of age-related macular degeneration. *Curr Opin Ophthalmol* 2009;20:151–157. 10.1097/ICU.0b013e32832af468 19390436

[21] Brahmi F, et al. Prevention of 7-ketocholesterol-induced side effects by natural compounds. *Crit Rev Food Sci Nutr* 2018:1–20. 10.1080/10408398.2018.1491828 29993272

[22] Dugas B, et al. Effects of oxysterols on cell viability, inflammatory cytokines, VEGF, and reactive oxygen species production on human retinal cells: cytoprotective effects and prevention of VEGF secretion by resveratrol. *Eur J Nutr* 2010;49:435–446. 10.1007/s00394-010-0102-2 20339855

[23] Sun W, Seigel GM, Salvi RJ. Retinal precursor cells express functional ionotropic glutamate and GABA receptors. *Neuroreport* 2002;13:2421–2424. 10.1097/00001756-200212200-00009 12499841

[24] Seigel GM. Review: R28 retinal precursor cells: the first 20 years. *Mol Vis* 2014;20:301–306. PMC3955414 24644404

[25] Dunn KC, et al. ARPE-19, a human retinal pigment epithelial cell line with differentiated properties. *Exp Eye Res* 1996;62:155–169. 10.1006/exer.1996.0020 8698076

[26] Moustafa MT, et al. Protective Effects of memantine on hydroquinone-treated human retinal pigment epithelium cells and human retinal muller cells. *J Ocul Pharmacol Ther* 2017;33:610–619. 10.1089/jop.2016.0129 28961056

[27] Lee JW, Huang JD, Rodriguez IR. *Extra-hepatic metabolism of 7-ketocholesterol occurs by esterification to fatty acids via cPLA2alpha and SOAT1 followed by selective efflux to HDL.* *Biochim Biophys Acta* 2015;1851:605–619. 10.1016/j.bbalip.2015.01.007 PMC4363286 25617738

[28] Rodriguez IR, et al. 7-ketocholesterol accumulates in ocular tissues as a consequence of aging and is present in high levels in drusen. *Exp Eye Res* 2014;128:151–155. 10.1016/j.exer.2014.09.009 PMC4254360 25261634

[29] Jang ER, Lee CS. 7-ketocholesterol induces apoptosis in differentiated PC12 cells via reactive oxygen species-dependent activation of NF-kappaB and Akt pathways. *Neurochem Int* 2011;58:52–59. 10.1016/j.neuint.2010.10.012 21035514

[30] Pedruzzi E, et al. NAD(P)H oxidase Nox-4 mediates 7-ketocholesterol-induced endoplasmic reticulum stress and apoptosis in human aortic smooth muscle cells. *Mol Cell Biol* 2004;24:10703–10717. 10.1128/MCB.24.24.10703-10717.2004 PMC533993 15572675

[31] Shimozawa M, et al. *7*-Ketocholesterol enhances the expression of adhesion molecules on human aortic endothelial cells by increasing the production of reactive oxygen species. *Redox Rep* 2004;9:370–375. 10.1179/135100004225006902 15720835

[32] Ong JM, et al. Oxysterol-induced toxicity in R28 and ARPE-19 cells. *Neurochem Res* 2003;28:883–891. 10.1023/a:1023223409798 12718442

[33] Casson RJ. Possible role of excitotoxicity in the pathogenesis of glaucoma. *Clin Exp Ophthalmol* 2006;34:54–63. 10.1111/j.1442-9071.2006.01146.x 16451260

[34] Heinen-Kammerer T, et al. Added therapeutic value of memantine in the treatment of moderate to severe Alzheimer's disease. *Clin Drug Investig* 2006;26:303–314. 10.2165/00044011-200626060-00001 17163264

[35] Kim TW, et al. Neuroprotective effect of memantine in a rabbit model of optic nerve ischemia. *Korean J Ophthalmol* 2002;16:1–7. 10.3341/kjo.2002.16.1.1 12162511

[36] Lipton SA, Rosenberg PA. Excitatory amino acids as a final common pathway for neurologic disorders. *N Engl J Med* 1994;330:613–622. 10.1056/NEJM199403033300907 7905600

[37] WoldeMussie E, et al. Neuroprotective effect of memantine in different retinal injury models in rats. *J Glaucoma* 2002;11:474–480. 10.1097/00061198-200212000-00003 12483089

[38] Chen B, et al. Memantine attenuates cell apoptosis by suppressing the calpain-caspase-3 pathway in an experimental model of ischemic stroke. *Exp Cell Res* 2017;351:163–172. 10.1016/j.yexcr.2016.12.028 28069373

[39] Ota H, et al. Protective effects of NMDA receptor antagonist, memantine, against senescence of PC12 cells: a possible role of nNOS and combined effects with donepezil. *Exp Gerontol* 2015;72:109–116. 10.1016/j.exger.2015.09.016 26408226

[40] Jantas-Skotniczna D, Kajta M, Lason W. Memantine attenuates staurosporine-induced activation of caspase-3 and LDH release in mouse primary neuronal cultures. *Brain Res* 2006;1069:145–153. 10.1016/j.brainres.2005.11.055 16386235

[41] Seigel GM, et al. Neuronal gene expression and function in the growth-stimulated R28 retinal precursor cell line. *Curr Eye Res* 2004;28:257–269. 10.1076/ceyr.28.4.257.27831 15259295

[42] Jantas D, et al. An involvement of BDNF and PI3-K/Akt in the anti-apoptotic effect of memantine on staurosporine-evoked cell death in primary cortical neurons. Apoptosis 2009;14:900–912. 10.1007/s10495-009-0370-6 19521778

[43] Dias IHK, et al. Simvastatin reduces circulating oxysterol levels in men with hypercholesterolaemia. *Redox Biol* 2018;16:139–145. 10.1016/j.redox.2018.02.014 PMC5952874 29501047

[44] Franke C, et al. Bcl-2 upregulation and neuroprotection in guinea pig brain following chronic simvastatin treatment. *Neurobiol Dis* 2007;25:438–445. 10.1016/j.nbd.2006.10.004 17157514

[45] Johnson-Anuna LN, et al. Simvastatin protects neurons from cytotoxicity by up-regulating Bcl-2 mRNA and protein.* J Neurochem* 2007;101:77–86. 10.1111/j.1471-4159.2006.04375.x 17241114

[46] Kretz A, et al. Simvastatin promotes heat shock protein 27 expression and Akt activation in the rat retina and protects axotomized retinal ganglion cells in vivo. *Neurobiol Dis* 2006;21:421–430. 10.1016/j.nbd.2005.08.003 16168661

[47] Miyahara S, et al. Simvastatin inhibits leukocyte accumulation and vascular permeability in the retinas of rats with streptozotocin-induced diabetes. *Am J Pathol* 2004;164:1697–1706. 10.1016/S0002-9440(10)63728-5 PMC1615657 15111316

[48] Muck AO, Seeger H, Wallwiener D. *C*lass-specific pro-apoptotic effect of statins on human vascular endothelial cells. *Z Kardiol* 2004;93:398–402. 10.1007/s00392-004-0081-5 15160275

[49] Urbich C, et al. Double-edged role of statins in angiogenesis signaling. *Circ Res* 2002;90:737–744. 10.1161/01.res.0000014081.30867.f8 11934843

[50] Weis M, et al. Statins have biphasic effects on angiogenesis. *Circulation* 2002;105:739–745. 10.1161/hc0602.103393 11839631

[51] Boucher K, et al. HMG-CoA reductase inhibitors induce apoptosis in pericytes. *Microvasc Res* 2006;71:91–102. 10.1016/j.mvr.2005.11.007 16427097

[52] Fujino M, et al. Counteracting effects of high density lipoprotein-cholesterol subfractions on statin-induced growth arrest. *Cardiovasc Drugs Ther* 2005;19:113–118. 10.1007/s10557-005-0427-x 16025229

[53] Krikorian R, et al. Concord grape juice supplementation improves memory function in older adults with mild cognitive impairment. *Br J Nutr* 2010;103:730–74. 10.1017/S0007114509992364 20028599

[54] Steffen Y, et al. Protein modification elicited by oxidized low-density lipoprotein (LDL) in endothelial cells: protection by (-)-epicatechin. *Free Radic Biol Med* 2007;42:955–970. 10.1016/j.freeradbiomed.2006.12.024 17349924

[55] Steffen Y, Schewe T, Sies H. Epicatechin protects endothelial cells against oxidized LDL and maintains NO synthase. *Biochem Biophys Res Commun* 2005;331:1277–1283. 10.1016/j.bbrc.2005.04.035 15883014

[56] Wang MH, et al. (-)-Epigallocatechin-3-gallate decreases the impairment in learning and memory in spontaneous hypertension rats. *Behav Pharmacol* 2012;23:771–780. 10.1097/FBP.0b013e32835a3bc8 23044831

[57] Zhang B, Osborne NN. Oxidative-induced retinal degeneration is attenuated by epigallocatechin gallate. *Brain Res* 2006;1124:176–187. 10.1016/j.brainres.2006.09.067 17084820

[58] Li S, et al. Characterization of the responses of the caspase 2, 3, 6 and 8 genes to immune challenges and extracellular ATP stimulation in the Japanese flounder (Paralichthys olivaceus). *BMC Vet Res* 2019;15:20. 10.1186/s12917-018-1763-y PMC6325855 30621683

[59] Li GY, Fan B, Su GF. Acute energy reduction induces caspase-dependent apoptosis and activates p53 in retinal ganglion cells (RGC-5). *Exp Eye Res* 2009;89:581–589. 10.1016/j.exer.2009.06.004 19524568

[60] Mansoor S, et al. Inhibition of apoptosis in human retinal pigment epithelial cells treated with benzo(e)pyrene, a toxic component of cigarette smoke. *Invest Ophthalmol Vis Sci* 2010;51:2601–2607. 10.1167/iovs.09-4121 19959636

[61] Kozlowski MR. The ARPE-19 cell line: mortality status and utility in macular degeneration research. *Curr Eye Res* 2015;40:501–509. 10.3109/02713683.2014.935440 24977298

